# Supplementary material for: Correlation Comparison and Personalized Utility of Field Walking Tests in Assessing the Exercise Capacity of Patients with Chronic Obstructive Pulmonary Disease: A Randomized Controlled Trial
Source: J Pers Med. 2022 May 30;12(6):901. doi: 10.3390/jpm12060901 (PMC9224606; doi:10.3390/jpm12060901)
Supplement: Supplementary file 1 [file jpm-12-00901-s001.zip › jpm-1708940-SI.pdf]

Online Supplement

Correlation comparison and personalized utility of field walking tests in assessing the exercise capacity of patients with chronic obstructive pulmonary disease: A randomized controlled trial

Eun Jae Ko[1*], Jang Ho Lee[2*], Hyang Yi Lee[3], Seong Ho Lee[3], Hack-Jae Lee[4], Ganghee Chae[5], Sei Won Lee[2#], Seung Won Ra[5#]

METHODS

Participants

Patients with COPD were recruited from May 2020 to December 2020 from the Department of Pulmonology, Ulsan University Hospital (Hospital 1) and Asan Medical Center (Hospital 2). The inclusion criteria were as follows: (i) patients were diagnosed with COPD; (ii) aged >40 years; and (iii) documented airflow limitation defined as post-bronchodilator forced expiratory volume in 1 second (FEV_1)/forced vital capacity (FVC) < 0.7. The exclusion criteria were as follows: (i) unstable angina or myocardial infarction within 1 month; (ii) uncontrolled arrhythmias causing symptoms or hemodynamic compromise, aortic dissection, history of syncope, left main coronary stenosis, hypertrophic cardiomyopathy, or acute endocarditis; (iii) acute pulmonary embolism, thrombosis of lower extremities, significant pulmonary hypertension, or pulmonary edema; (iv) acute respiratory failure; (v) resting heart rate (HR) >120; (vi) resting saturation of percutaneous oxygen (SpO_2) <85; (vii) patients who need oxygen therapy when walking; (viii) severe untreated arterial hypertension at rest (180 mmHg systolic, 100 mmHg diastolic); (ix) acute noncardiopulmonary disorder that may affect exercise performance or be aggravated by exercise (i.e., infection, renal failure, or thyrotoxicosis); (x) pregnancy; (xi) electrolyte abnormalities; and (xii) orthopedic impairment that prevents

walking. We reviewed the information about height, weight, body mass index, smoking history, comorbidity, history and degree of COPD aggravation, modified Medical Research Council (mMRC) dyspnea scale, COPD Assessment Test (CAT), results of chest X-ray, results of chest computed tomography, results of pulmonary function test (PFT), such as forced vital capacity (FVC), forced expiratory volume in 1 second (FEV_1), FEV_1/FVC, forced expiratory flow at 25%–75% (FEF25%–75%), results of echocardiography, results of echocardiogram, and medication.

Table S1. Comparison of the correlation between peak oxygen uptake and distance of ISWT and 6MWT without four patients with higher breathing reserve

	**Total** (*n* = 25)		**Hospital 1** (*n* = 14)		**Hospital 2** (*n* = 11)		P^*
	r	*P*	*r*	*P*	*r*	*P*	
PeakVO$_2$ ~ ISWT	0.783	<0.001	0.867	<0.001	0.643	0.033	0.230
PeakVO$_2$ ~ 6MWT	0.620	0.001	0.679	0.008	0.754	0.007	0.739
$P^{\dagger}$	0.208		0.037		0.600		

Correlation coefficient (r) was analyzed using Pearson's correlation.

*P value was analyzed using the Fisher's z transformation; †P value was analyzed using the Dunn and Clark's z test.

Abbreviations: ISWT, incremental shuttle walking test; 6MWT, 6-min walking test; peak VO2, peak oxygen consumption.

Table S2. Clinical parameters of each exercise test of the total study population

	**ISWT** (n = 29)	**6MWT** (n = 29)	**CPET** (n = 29)	P
Peak SBP	159.0 (146.5–175.5)	151.0 (132.5–165.0)	193.0 (164.5–216.5)	<0.001
Peak DBP	89.0 (81.5–95.5)	85.0 (74.5–88.5)	95.0 (83.5–106.0)	0.004
Peak HR	118.0 (110.0–136.0)	110.0 (96.0–124.0)	130.0 (119.5–142.5)	0.001
Lowest SpO_2	92.0 (86.5–94.0)	94.0 (89.5–96.0)	92.0 (89.0–94.0)	0.194
Pre Borg score	0.5 (0.0–1.0)	0.0 (0.0–0.0)	0.0 (0.0–0.0)	<0.001
Post Borg score	5.0 (4.0–5.5)	3.0 (1.0–4.0)	10.0 (7.0–10.0)	<0.001

The values were presented as median (interquartile range), and P value was analyzed using the Kruskal–Wallis U test.
Abbreviations: 6MWT, 6-min walking test; CPET, cardiopulmonary exercise test; DBP, diastolic blood pressure; HR, heart rate; ISWT, incremental shuttle walking test; peak VO_2, peak oxygen consumption; SBP, systolic blood pressure; SpO_2, oxygen saturation

Table S3. Clinical parameters at each exercise tests of two hospitals

	Hospital 1 (Ulsan University Hospital)				Hospital 2 (Asan Medical Center)			
	ISWT	6MWT	CPET	*P*	ISWT	6MWT	CPET	*P*
Peak SBP	150.0 [142.5–167.0]	130.5 [124.0–152.0]	169.0 [153.0–199.0]	0.001	166.5 [150.3–184.5]	162.0 [148.5–172.3]	215.5 [188.8–220.0]	<0.001
Peak DBP	84.5 [77.0–95.5]	74.5 [63.5–83.5]	85.0 [71.0–100.0]	0.029	90.0 [88.0–96.5]	88.0 [85.0–92.0]	102.5 [94.8–118.5]	0.001
Peak HR	123.0 [109.0–140.5]	108.5 [96.5–121.0]	131.0 [109.0–148.0]	0.011	112.0 [109.0–136.0]	110.5 [91.0–122.8]	130.0 [120.3–138.8]	0.044
Lowest SpO_2	89.0 [83.5–92.5]	94.0 [88.5–95.5]	91.0 [88.0–94.0]	0.286	93.0 [86.5–96.3]	94.5 [90.0–96.3]	93.0 [89.8–94.3]	0.645
Pre Borg score	1.0 [0.5–2.0]	0.0 [0.0–0.0]	0.0 [0.0–0.0]	<0.001	0.0 [0.0–0.0]	0.0 [0.0–0.0]	0.0 [0.0–2.3]	0.014
Post Borg score	4.0 [4.0–5.0]	1.0 [0.5–3.0]	10.0 [10.0–10.0]	<0.001	5.0 [4.0–7.5]	4.0 [3.0–5.0]	7.0 [6.0–7.5]	<0.001

The values were presented as median (interquartile range), and *P* value was analyzed using the Kruskal–Wallis *U* test.
Abbreviations: 6MWT, 6-min walking test; CPET, cardiopulmonary exercise test; DBP, diastolic blood pressure; HR, heart rate; ISWT, incremental shuttle walking test; peak VO_2, peak oxygen consumption; SBP, systolic blood pressure; SpO_2, oxygen saturation

Figure Legends

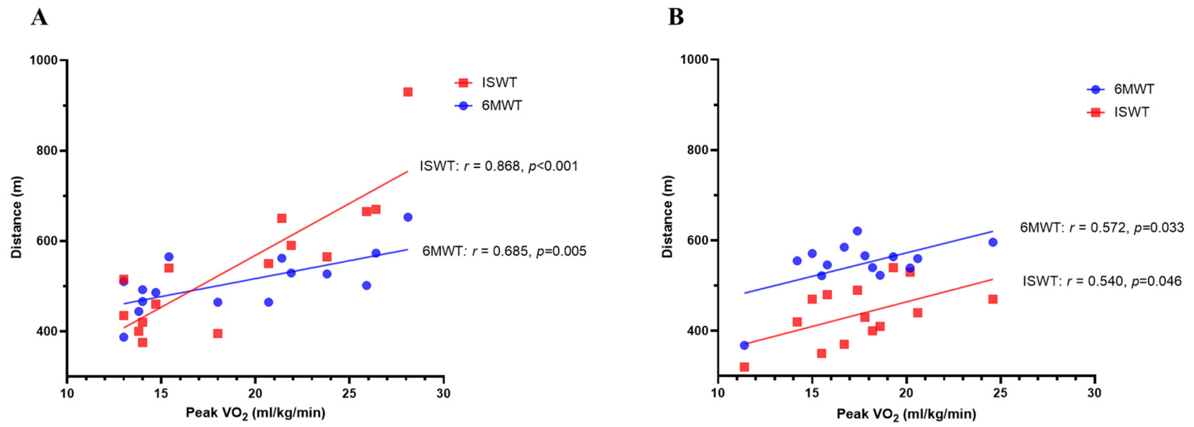

Figure S1. The correlation analysis of ISWT and 6MWT in each hospital. The validity of ISWT and 6MWT was analyzed using the Pearson's correlation coefficient (*r*). A, Hospital 1 (Ulsan University Hospital). B, Hospital 2 (Asan Medical Center). Abbreviations: 6MWT, 6-minute walking test; ISWT, incremental shuttle walking test; Peak VO_2, peak oxygen consumption.

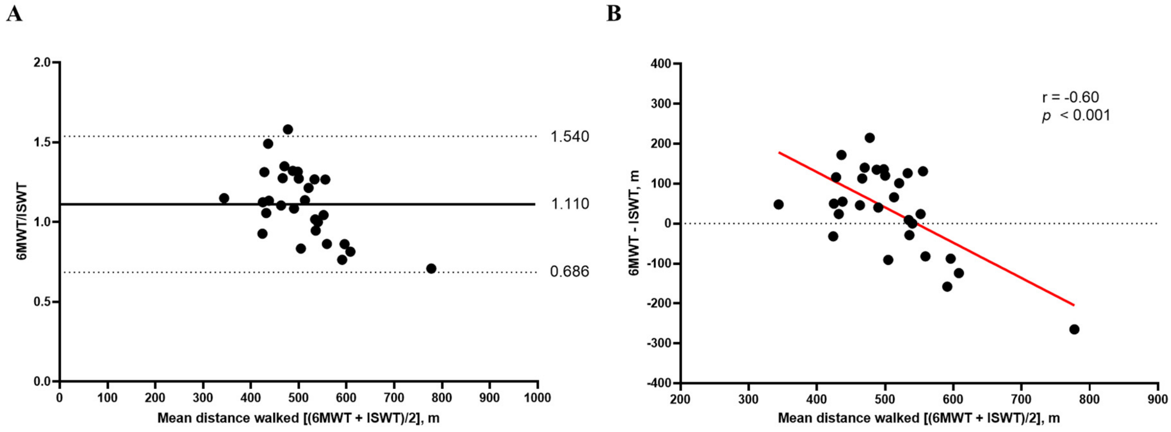

Figure S2. The correlation analysis between ISWT and 6MWT. A, Bland–Altman plot of the ratio of the walking distance on 6MWT and ISWT. Patients walked 11% ± 43% more in the 6MWT than in ISWT. B, the correlation analysis between 6MWT–ISWT differences on distance (6MWT–ISWT) and the average walked distance. The correlation coefficient (*r*) was analyzed using the Pearson's correlation. Patients, who walked lower mean distance, presented

the higher 6MWT– ISWT. Abbreviations: 6MWT, 6-minute walking test; ISWT, incremental shuttle walking test.
